# Assessment and management of pulmonary alveolar proteinosis in a reference center

**DOI:** 10.1186/1750-1172-8-40

**Published:** 2013-03-13

**Authors:** Ilaria Campo, Francesca Mariani, Giuseppe Rodi, Elena Paracchini, Eric Tsana, Davide Piloni, Isabella Nobili, Zamir Kadija, Angelo Corsico, Isa Cerveri, Claudia Chalk, Bruce C Trapnell, Antonio Braschi, Carmine Tinelli, Maurizio Luisetti

**Affiliations:** 1 Respiratory Disease Unit, Fondazione IRCCS Policlinico San Matteo, University of Pavia, Pavia, Italy; 2 Anesthesia and Intensive Care 1, Fondazione IRCCS Policlinico San Matteo, University of Pavia, Pavia, Italy; 3 Cincinnati Children’s Hospital Medical Center, Cincinnati, OH, USA; 4 Clinical Epidemiology and Biometric Unity, Fondazione IRCCS Policlinico San Matteo, Pavia, Italy

## Abstract

Pulmonary alveolar proteinosis (PAP) is a term defining an ultra-rare group of disorders characterised by a perturbation in surfactant homeostasis, resulting in its accumulation within airspaces and impaired gas transfer. In this report we provide data from a cohort of PAP patients (n = 81) followed for more than two decades at the San Matteo University Hospital of Pavia, Italy. In agreement with other large series in PAP individuals, 90% of the study subjects were affected by autoimmune/idiopathic PAP, while the remaining subjects were divided as follow: congenital 1%, secondary 4% and PAP-like 5%. The disease affected males and females with a ratio of 2:1 and approximately one third of PAP patients were lifelong nonsmokers. Occupational exposure was reported in 35% of subjects in this series. With reference to the PAP clinical course, in 29 patients (7% with spontaneous remission) disease severity did not necessitate whole lung lavage (WLL) in the long-term follow up. On the other hand, 44 PAP patients underwent therapeutic WLL: in 31 subjects a single WLL was sufficient to provide long term, durable benefit, whereas 13 patients required multiple WLLs. The intra-patient mean interval between two consecutive WLLs was 15.7 ± 13.6 months. When baseline data among never lavaged and PAP patients lavaged at least once were compared, the need for lavage was significantly associated with serum biomarkers (CEA, Cyfra, LDH), lung function parameters forced vital capacity (FVC), and lung diffusing capacity (Dlco). We conclude that patient cohorts with an ultra-rare disease, such as PAP, referred to a single reference center, can provide useful information on the natural history and clinical course of the disease.

## Background

Pulmonary alveolar proteinosis (PAP) is a syndrome characterized by the accumulation of surfactant within alveolar macrophages and alveoli, which impairs pulmonary gas transfer and results in clinical severity ranging from an asymptomatic clinical presentation to respiratory failure and death [1]. The prevalence of autoimmune (previously referred to as idiopathic) PAP is 0.1/100,000 , and accounts for about 90% of PAP cases [2,3]. According to such figures, PAP is considered an ultra-rare disorder. In spite of major advances achieved in the late ‘90s when the group of Koh Nakata first described the presence of autoantibodies neutralizing GM-CSF in serum and lung tissue of patients with idiopathic PAP [4,5], several specific treatments have been attempted or postulated [6], but the standard of care is still the whole lung lavage (WLL), eventually modified after the original description by Ramirez in 1963 [7]. As a result, nowadays PAP is no longer considered a potentially lethal disorder.

Descriptions of large series of PAP patients in the literature over the years have greatly extended the knowledge on the natural history of the disorder. Following the meta-analysis of 410 cases by Seymour and Presneill [8], who reviewed all PAP cases available in the literature, including 34 PAP cases followed at the Mayo Clinic Center in Rochester [9], other large multicenter series were published in Japan in 2008 (248 cases) [10] and China (241 cases) [11]. In 2011, Bonella and associates described the characteristics of a cohort of 70 PAP patients followed at a single center in Essen, Germany [12]. In this communication we report on a cohort of 81 PAP patients registered at the Pneumology Section of the San Matteo University Hospital of Pavia, Italy. It should be emphasized that the series of patients described in a single reference center, although less relevant than large meta-analysis or multicenter series, has however the advantage of including detailed experience accumulated over several years (decades, as in our case).

This is of special relevance as many treatment aspects, such as WLL for PAP, have not been investigated in depth.

In this report, we have focused on two aims : first, the description of the clinical characteristics of the disease at presentation in our series of PAP patients; secondly, to analyse some baseline functional and biochemical parameters in function of the long term follow up of patients, especially with respect to the need for WLL. The latter has not been covered in the literature so far.

## Methods

### Patients’ diagnosis and assessment

Progressive recruitment of patients with surfactant associated disorders started after the first WLL performed in 1989. Diagnostic tools were the presence of “crazy paving pattern” at the high resolution computed tomography scan images of the thorax and the presence of macroscopic milky fluid and/or the presence of amorphous, eosinophilic, PAS positive material, as well as lipid laden macrophages on bronchoalveolar lavage analysis [1].

Determination of serum level of autoantibodies anti-GM-CSF (GMAbs) was performed in the Laboratory of the Rare Lung Disease Consortium at the Cincinnati Children’s Hospital Medical Center beginning 2004 [4,5]. Lung biopsies (surgical or transbronchial), if available, were reviewed and evaluated according to current criteria for diagnosis of PAP [13]. Diagnosis of the kind of surfactant associated disorder was performed according to the classification: primary (also referred to as idiopathic or autoimmune) PAP, secondary PAP, hereditary PAP, and PAP-like disorders [1]. Pulmonary functional assessment and WLL were performed according to Beccaria and coworkers [14]. The indication for WLL were as follows : a) persistent or progressive respiratory failure; b) no respiratory failure, but drop by 5 or more percentage points of O2 saturation on treadmill exercise (modified Bruce protocol) determined by pulse oxymetry [14]. Serum measurements described in the text (LDH, Cyfra 21-1, CEA, NSE) were performed in the clinical chemistry facilities according to internal standard operative procedures.

The investigation was conducted in compliance with the Helsinki declaration and approved by the Ethics Committee of the San Matteo Hospital Foundation.

### Statistical analysis

The Shapiro-Wilk test was used to test the normal distribution of quantitative variables. When quantitative variables were normally distributed, the results were expressed as mean values and SD, otherwise median and interquartile range (IQR; 25^th^ -75^th^ percentile) were reported. The one-way ANOVA (or non parametric Kruskall-Wallis for skewed distributions), with Bonferroni correction for comparisons between two groups, was employed to investigate differences among the three study groups (PAP patients never lavaged, lavaged once or more times). Qualitative variables were summarized as counts and percentages and the Chi-square (χ2) test were used to compare gender among study groups. P < 0.05 was considered statistically significant and all tests were two-sided.

Data analysis was performed with the STATA statistical package (release 11,1, 2010, Stata Corporation, College Station, Texas, USA).

## Results

**Figure 1:**
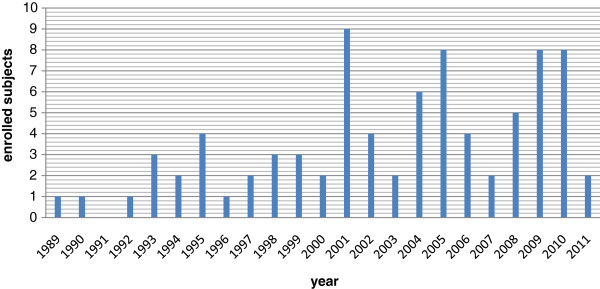
Progressive enrollment of subjects with surfactant associated disorders in the Pavia center.

Beginning January 1989, we progressively evaluated subjects admitted to our center with surfactant associated disorders (Figure 1). As of June 2011, eighty-one patients had been enrolled and classified according to Luisetti & Trapnell [1] (Tables 1 and 2). In order to keep the study group as homogeneous as possible, in this paper we focused on primary PAP (idiopathic and autoimmune) patients only, herein collectively referred to as PAP patients. Demographic, clinical, and assessment features of the 73 PAP patients are reported in Table 2. The male:female ratio was 2:1. With reference to environmental factors, the majority of PAP patients in our series were smokers (either current or former), but a significant fraction of subjects (33%) never smoked. Among those reporting professional exposure, 19 out of 26 were exposed to inorganic dusts (silica, cement), 1 to organic dusts, 2 to combustion products, 1 to chemicals (photographic fixer) and 3 to pesticides.

**Table 1:** Demographics of the 81 subjects with surfactant associated disorders

	**n.**	**%**
**Classification**		
Primary PAP		
Idiopathic + autoimmune^1^	73	90
Hereditary^2^	1	1
Secondary PAP^3^	3	4
PAP-like^4^	4	5
**Gender**		
Male	54	66.5
Female	27	33.5
**Smoking status**		
Current	18	22
Former	34	42
Never	29	36

^1^ We began to measure serum GMAb level in 2004, thus for accuracy in this group we included patients with *idiopathic PAP* (n = 42, those diagnosed before 2004) and patients with *autoimmune PAP* (n = 31, those diagnosed after 2004), although currently they are considered synonymous. ^2^ This subject was affected by PAP due to a GM-CSF receptor αchain mutation [17]. ^3^ This group includes two subjects with PAP secondary to hematologic disorders (1 chronic myeloid leukemia and 1 myelodysplastic syndrome) and 1 subject affected by lysinuric protein intolerance (the latter’s case history has already been reported) [18,19]. ^4^ This group includes four subjects with a mutation in the ABCA3 gene (manuscript in preparation).

**Table 2:** Characteristics and assessment of the PAP cohort

	**n.**	**%**	**m ± sd**
	**73**		
**Age**			41 ± 12.8 (yrs)
**Gender**			
Male	52	71	
Female	21	29	
**Smoking status**			
Current	16	22	
Former	33	45	
Never	24	33	
**Exposures**^1^	26	36	
**Symptoms at enrollment**			
Symptomatic	69	95	
dyspnea^2^	49	67	
cough	23	31	
fever	8	11	
sputum production	1	1	
Asymptomatic	4	5	
**Diagnostic procedures**^3^	69		
HR CT Scan	51	74	
Biopsy	41	56	
BAL	32	46	
GMAbs^4^	31	45	
**Infection at enrollment**^4^	14	19	
**Lung Function**^**5**^			
FVC	57		75.50 ± 21.1 (% predicted)
FEV_1_	57		77.77 ± 18.49 (% predicted)
TLC	57		75.38 ± 14.77 (% predicted)
DLCO	57		51.83 ±17.90 (% predicted)
PaO2	57		64.46 ± 14.17 (mm Hg)
ΔA-aO2	57		41.44 ± 16.25 (mm Hg)
Exercise Sat.O2	57		-4.27% ± 1.93
**Biomarkers **^**6**^			
Cyfra 21-1	26		12.8 ± 14.6 (ng/mL) (nv: 0.0-3.3)
CEA	27		13.4 ± 13.4 (ng/mL) (nv: 0.0-5.0)
NSE	23		20.04 ± 6.9 (ng/mL) (nv: 0.0-15.0)
LDH	57		550.3 ± 248 (U/L) (nv: 230-460)
GMAbs	31		184.1 ± 175 (μg/ml) (nv < 3)
**DSS **^**7**^	39		
1	2	5	
2	12	31	
3	13	33	
4	4	10	
5	8	21	

^1^Subjects exposed for work-related reasons to dust, smoke or gas of organic or inorganic origin. ^2^Patients may report more than one symptom.^3^No data available in 4/73 pts. More than one diagnostic tool was applied in 58 PAP patients (84%). Measurement of GMAbs began in 2004.^4^These include respiratory infections in 12 patients (4 *Staph.aureus*, 3 *Str.pneumoniae*, 1 *Pn.jiroveci*, 1 *Kleb.pneumoniae*, 1 *Nocardia ast*., 1 *Serratia Marcesc*., 1 *Candida Alb*) and 2 isolates of *H.pilorii* from gastric biopsy. ^5^Abbreviation legends: FVC: forced vital capacitiy; FEV1: forced expiratory flow in the first second; TLC: total lung capacity; DLCO: diffusing lung capacity for carbon monoxide; PaO2: arterial oxygen tension; ΔA-aO2: alveolar to arterial oxygen tension difference;%predicted: percentage of the predicted value; nv: normal value. ^6^Abbreviation legends: Cyfra 21-1: cytokeratin 19 fragment 21-1; CEA: carcinoembrionic antigen; NSE: neuron specific enolase; LDH: lactate dehydrogenase. ^7^ DSS: disease severity score [10].

Among symptoms reported at the time of diagnosis, the majority of PAP patients complained about dyspnea (67%) and cough (31%); only one reported sputum production.

Among the four diagnostic tools used, 74% of patients were assessed by high resolution (HR) CT scan, followed by lung biopsy (surgical or transbronchial), and bronchoalveolar lavage (BAL). Interestingly, the introduction of GMAbs measurement in 2004 did not substantially change the diagnostic attitude: before 2004, lung biopsy was performed in 60.6% of PAP patients, and thereafter the percentage dropped only to 52.5%. Twelve patients were affected by a respiratory infection at the moment of diagnosis. As far as the lung function data are concerned, our results are in line with the previous functional report on our initial PAP series [14], with relevant impairment particularly in DLco and Δ(A-a)O2.

The mean value of all available biomarkers were above the reference upper limit; the GMAbs measurement, became available in 2004, confirmed the autoimmune nature in 100% of PAP cases diagnosed since that year. Finally, about two thirds of the PAP patients were classified as Disease Severity Score (DSS) 2 or 3 [10,15,16].

All patients from our cohort were evaluated for WLL, the current standard of care for PAP.

**Figure 2:**
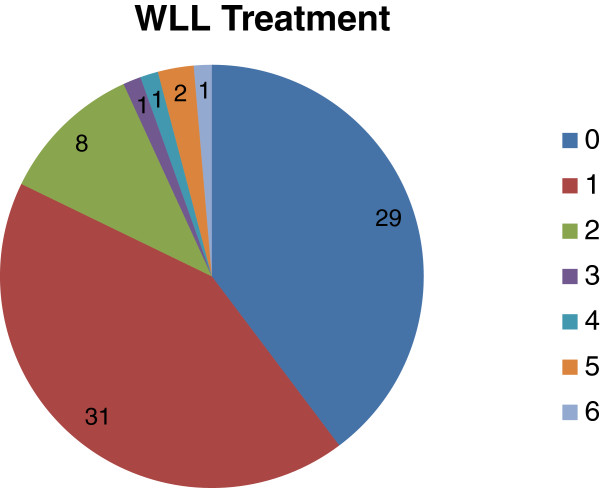
**PAP patients divided according to the number of WLLs received.** Twenty-nine patients were never submitted to WLL, whereas 44 were lavaged at least once. PAP patients requiring an additional WLL within 30 days from the previous one (3/31), were considered to be submitted to a single WLL. In patients requiring multiple WLLs, the mean (± SD) interval between two consecutive WLLs was 15.7 (±13.7) months.

According to above described criteria, 44 out of 73 PAP patients (60%) were submitted to 1 or multiple WLLs, whereas 29 patients (40%) did not require WLL for management of their respiratory symptoms (Figure 2). Baseline data between never lavaged PAP patients were compared with those who had undergone one or repeated lavages and are reported in Table 3. The analysis of the data available from 28 out of 44 lavaged PAP patients are presented; in the remaining 16 PAP patients the data set was incomplete, mostly because they were referred to our center or directly to the Intensive Care Unit in such a severe that a complete pre-lavage functional assessment was not possible. Interestingly, this analysis thus refers to a relatively mildly affected subset of PAP patients, in which the decision to perform WLL was sometimes more difficult. According to these data, the best predictors for the need to perform WLL were biomarkers (CEA, Cyfra, LDH) and the functional parameters diffusing capacity (DLco) and FVC. Interestingly, the disease severity score (DSS), combining presence or absence of symptoms with level of PaO2, was not significantly different between not lavaged and lavaged PAP patients. Consistently, also the Δ(A-a)O2 and PaO2 did not significantly differ. No baseline parameter was able to discriminate PAP patients requiring in their follow up 1 single WLL from those eventually requiring more than 1 WLLs.

**Table 3:** Analysis of baseline pulmonary function data between PAP patients never lavaged and lavaged once or more times

		**No WLL (n = 19)**	**1 WLL (n = 18)**	**>1 WLL (n = 10)**	**p value**
CEA	median (iqr)	5 (3-8)	13 (8-30)	8.5 (7-12)	**0.0418019**^**1**^
Cyfra	median (iqr)	4.5 (2-5)	13 (9-16)	5 (2-32.5)	**0.0275304**^**2**^
LDH	mean (sd)	368.67 (109.78)	622.14 (263.26)	536.2 (166.89)	**0.0126793**^**3**^
NSE	mean (sd)	19.2 (5.72)	20.6 (6.22)	22 (4)	0.8022085
DSS	mean (sd)	2.67 (1.22)	3.06 (1.25)	3.57 (.98)	0.3353056
FEV1 (%Pred)	mean (sd)	82.82 (14.79)	75.8 (22.63)	65.88 (12.23)	0.1087558
FEV1/Vc (%Pred)	mean (sd)	100.43 (5.17)	105.47 (8.71)	103 (8.25)	0.1804334
FVC (%Pred)	mean (sd)	83.89 (13.16)	73.17 (21.83)	61.57 (9.03)	**0.0145088**^**4**^
PaCo2 (mmHg)	mean (sd)	35.15 (3.4)	33.91 (3.9)	33.93 (4.07)	0.6664365
PaO2 (mmHg)	mean (sd)	70.95 (13.25)	62.64 (13.01)	59.2 (8.96)	0.1019723
TLC (%pred)	mean (sd)	79.43 (8.15)	74.47 (17.92)	65.43 (14.03)	0.115102
DLco (%pred)	mean (sd)	61.89 (19.27)	45.95 (14.29)	42.63 (11.17)	**0.0038198**^**5**^
DLco (mmolkpas)	mean (sd)	6.52 (3.41)	4.08 (1.61)	3.84 (1.06)	**0.0087515**^**6**^
ΔA-aO2	mean (sd)	34.85 (16.28)	44.84 (16.43)	48.14 (8.76)	0.1299473

^1^no WLl vs 1 WLL: p = 0.006057; ^2^no WLl vs 1 WLL: p = 0.005202; ^3^no WLl vs 1 WLL: p = 0.011; ^4^no WLl vs >1 WLL: p = 0.016; ^5^no WLl vs 1 WLL: p = 0.010, no WLl vs >1 WLL: p = 0.021; ^6^no WLl vs 1 WLL: p = 0.015, no WLl vs >1 WLL: p = 0.05.

## Discussion

In this study, the enrollment characteristics of the largest series of PAP patients followed in a single, reference center are described. In spite of the retrospective nature of the study, the series of PAP patients was large enough to evaluate some selected parameters as indicators of WLL.

In a previous study [14] we presented baseline data from an initial cohort and assessed the long-term efficacy of WLL as the current standard of care for PAP [5]. In that study, a total of 21 PAP patients were evaluated, of whom 15 agreed to participate in a long-term follow up after WLL. In the present paper, after 11 years of progressive recruitment, we present data from a total of 73 patients with autoimmune/idiopathic PAP. To increase the impact of our findings, we discuss the enrollment data in comparison, when possible, with those of other large series of PAP patients [8,10-12] (Table 4). This table includes data from a total of 1,050 PAP patients, the largest group of PAP patients ever analyzed. We confirm that PAP is a disease affecting young adults (mean age ranging from 39 to 51 years), mostly males: the male:female ratio was the highest in the Seymour worldwide series [8] and the lowest in German patients described by Bonella [12]. There was a great agreement on the prevalence of secondary PAP cases : 90% of PAP cases are primary (or autoimmune) in the Japanese, Germans, and Italians, with a rate of 10% or less for secondary PAP [17-19]. Moreover, in all except the Chinese series [11] (which was not evaluated) the rate of spontaneous remitters is very homogeneous, ranging from 5 to 7% total cases, a much lower rate than that proposed in the past (about 30%) [9]. Data from the Italian PAP cohort follow the other series’ smoking habits: although a majority of PAP patients are smokers (current or former) a significant portion of PAP patients have never smoked, ranging from 21% in the German series to 43% in the Japanese cohort. Professional exposure was another feature with a possible impact on PAP pathogenesis [10]. It occurs at a variable rate: in our series it is similar to that of the Japanese (32 and 26%, respectively) and at a lower rate than in German series (54%). A marked difference among series was the rate of asymptomatic PAP cases at diagnosis: in our hands they account for 5% of the entire series, whereas, in the Japanese series they account for approximately 30% [10]. Another interesting finding, restricted to our series, is the elevation of cancer biomarkers (CEA, Cyfra 21-1, NSE) (Table 2). Although some papers have previously reported such an elevation [20,21], data were not sufficient to analyze such changes in detail and in longitudinal perspective, but this should be the aim of a future analysis.

**Table 4:** Comparison of published cohorts

	**Seymour (2002) **[8]** (n = 410)**	**Inoue (2008) **[10]** (n = 248)**	**Xu (2009) **[11]** (n = 241)**	**Bonella (2010) **[12]** (n = 70)**	**Campo (2012) [this paper] (n = 81)**
**Age at Diagnose** (mean, range)	39 (30-46)	51 (41-58)	42 (n.a)	43 (18-78)	40 (26-54)
**Ratio Male/Female**	2.6	2.0	2.2	1.3	2.0
**Primary PAP** (%)	n.a.	90	n.a.	91	90
**Secondary PAP** (%)	n.a.	10	n.a.	9	3.7
**Time to diagnosis** (months)	7 (3-19)	10 (4-36)	n.a.	9 (1-36)	11 (0-27)
**Spontaneous remitters** (%)	6	5	n.a.	5	7
**Smoking habits** (%)					
Never	28	43	-	21	36
Previous	n.a.	29	-	30	42
Current	n.a.	29	-	49	22
**Dust exposure (%)**	n.a.	26	n.a.	54	32
**Whole lung lavage**	54%	n.a.	59%	90%	54%

As emphasized above, WLL is the current standard of care for autoimmune and other forms of PAP [6]. As shown in Table 4, the procedure was adopted in 54% of PAP patients in the Seymour study, data are in line with the Seymour and Chinese series, with 54% of PAP patients submitted to WLL. Twenty-nine out of our 73 patients were never submitted to WLL during their follow up. No patient refused the treatment; the decision was based on medical evaluation, since the degree of lung involvement did not alter the lung function. In other words, in these patients the WLL, an invasive and potentially harmful procedure, was considered unnecessary. Of the 44 patients in our series submitted to WLL, in 31 (70%), a single WLL was sufficient to ensure long-term, durable benefit in lung function. Such a benefit, of course, does not exclude disease relapse, but not to such a severe degree requiring an additional WLL. It is noteworthy, that among the 31 PAP patients lavaged only once, included are 3 PAP patients who required two consecutive WLLs within one month, as no substantial improvement after the first lavage was achieved. In this case, the 2 consecutive WLLs were considered as one. These data confirm that in 2/3 of PAP patients, a single WLL is sufficient to induce long-term, and in some cases definitive, stable, improvement of lung function. In 13 PAP patients, multiple WLLs were necessary to maintain satisfactory lung function, and only in 5 out of 13, were more than 2 WLLs necessary. The mean interval between two WLLs in the same PAP patient was 15.7 ± 13.6 months.

Although performed in several centers, the WLL procedure still lacks standardization. Indications when to perform WLL remain one of the undefined aspects, since precise guidelines on patients who will benefit from WLL are still lacking. The huge difference among centers concerning the percentage of PAP patients submitted to WLL reported in Table 4 is a clear example of lack of precise guidelines. Respiratory failure, particularly when severe enough to require mechanical ventilation, does not need any decisional discussion, whereas latent respiratory failure, triggered by exercise only, with radiology imaging stable over a prolonged period of time, raises concerns on the decision to lavage. Although our data were unable to provide a definitive answer to this question, we endeavored to compare the baseline value of never lavaged PAP patients with those of PAP patients submitted to WLL (Table 3). Our aim was to evaluate some selected and available parameters with the Disease Severity Score (DSS), a combination of presence and absence of symptoms and degree of PaO2 [10,15], in the decision making whether or not to submit a patient to WLL. DSS was unable to discriminate PAP not requiring WLL from those mildly affected, with latent respiratory failure, whereas other parameters, such as the cancer biomarkers, FVC and DLco were significantly different in the PAP two populations. Consistently with the DSS behavior, PaO2 and Δ(A-a)O2 did not differ significantly. We therefore conclude that DSS is not an useful tool for decision making with respect to WLL, and we hypothesize that the score should be integrated with other parameters, such as those herein evaluated, and perhaps with a CT scoring system, to generate a composite marker. Hopefully such score could more useful to help in the decision for WLL and in the evaluation of the effectiveness of interventional trials. None of the tested markers was able to discriminate the PAP patients lavaged once from those requiring more than one single WLL.

A major limitation of the study is its retrospective nature, in turn affecting in some cases quality and availability of the collected data. This is however balanced by the remarkable sample size, taking into consideration the extreme rarity of PAP.

As already emphasized by Bonella and associates [12] we would like to reinforce the importance of gathering patients affected by this rare condition in a single reference center, where optimal care can be provided. This is especially the case with WLL, a procedure that has been performed for 50 years, first described in 1963 [7], but as yet not standardized. Moreover, the WLL procedure is seldom described in detail in the scientific literature. We believe that efforts should be increased to create an operating network among centers performing WLL either in adults or pediatric PAP patients, to share experience and develop common protocols to answer the many unanswered questions about WLL in PAP.

## Competing interests

The authors declare that they have no competing interests.

## Authors’ contributions

IC collected data and drafted the manuscript; CT interpreted, analyzed data and drafted the manuscript; FM, EP, ZK coordinated clinical diagnosis, blood collection and storage; IC and AC coordinated pulmonary function testing; GR, AB and FM performed WLL reatment; IN, IP, ET and DP were involved in clinical diagnosis and blood collection; CC and BCT performed autoantibody measurement; ML drafted and critically revised the manuscript and supervised the research. All authors read and approved the final manuscript.

## References

[B1] Luisetti M Trapnell BC Schwarz MI, King TE Pulmonary alveolar proteinosis Interstitial Lung Disease 2010 Shelton CT: Peoples’ Medical Publishing House USA 1079 1093

[B2] Trapnell BC Whitsett JA Nakata K Pulmonary alveolar proteinosis N Engl J Med 2003 349 26 2527 2539 10.1056/NEJMra023226 14695413

[B3] Prevalence of rare diseases Bibliographic data, Orphanet Report Series, Rare diseases collection November 2011, Number 1: Listed in alphabetical order of diseases http://www.orpha.net/orphacom/cahiers/docs/GB/Prevalence_of_rare_diseases_by_alphabetical_list.pdf

[B4] Kitamura T Tanaka N Watanabe J Uchida K Kanegasaki S Yamada Y Nakata K Idiopathic pulmonary alveolar proteinosis as an autoimmune disease with neutralizing antibody against granulocyte/macrophage colony-stimulating factor J Exp Med 1999 190 6 875 880 10.1084/jem.190.6.875 10499925 PMC2195627

[B5] Tanaka N Watanabe J Kitamura T Yamada Y Kanegasaki S Nakata K Lungs of patients with idiopathic pulmonary alveolar proteinosis express a factor which neutralizes granulocyte-macrophage colony stimulating factor FEBS Lett 1999 442 2–3 246 250 9929010 10.1016/s0014-5793(98)01668-8

[B6] Luisetti M Kadija Z Mariani F Rodi G Campo I Trapnell BC Therapy options in pulmonary alveolar proteinosis Ther Adv Respir Dis 2010 4 239 248 10.1177/1753465810378023 20647242

[B7] Ramirez J Schultz R Dutton R Pulmonary alveolar proteinosis: a New technique and rationale for treatment Arch Intern Med 1963 112 419 431 10.1001/archinte.1963.03860030173021 14045290

[B8] Seymour JF Presneill JJ Schoch OD Therapeutic efficacy of granulocyte macrophage colony-stimulating factor in patients with idiopathic acquired alveolar proteinosis Am J Respir Crit Care Med 2001 163 524 531 11179134 10.1164/ajrccm.163.2.2003146

[B9] Prakash UB Barham SS Carpenter HA Dines DE Marsh HM Pulmonary alveolar phospholipoproteinosis: experience with 34 cases and a review Mayo Clin Proc 1987 62 6 499 518 10.1016/S0025-6196(12)65477-9 3553760

[B10] Inoue Y Trapnell BC Tazawa R Arai T Takada T Hizawa N Kasahara Y Tatsumi K Hojo M Ichiwata T Tanaka N Yamaguchi E Eda R Oishi K Tsuchihashi Y Kaneko C Nukiwa T Sakatani M Krischer JP Nakata K Japanese center of the rare lung diseases consortium Characteristics of a large cohort of patients with autoimmune pulmonary alveolar proteinosis in japan Am J Respir Crit Care Med 2008 177 7 752 762 10.1164/rccm.200708-1271OC 18202348 PMC2720118

[B11] Xu Z Jing J Wang H Xu F Wang J Pulmonary alveolar proteinosis in China: a systematic review of 241 cases Respirology 2009 14 5 761 766 10.1111/j.1440-1843.2009.01539.x 19476601

[B12] Bonella F Bauer PC Griese M Ohshimo S Guzman J Costabel U Pulmonary alveolar proteinosis: new insights from a single-center cohort of 70 patients Respir Med 2011 105 12 1908 1916 10.1016/j.rmed.2011.08.018 21900000

[B13] Morbini P Guddo F Contini P Luisetti M Schiavina M Zompatori M Rare diffuse diseases of the lung. Pulmonary alveolar proteinosis, lymphangioleiomyomatosis, amyloidosis Pathologica 2010 102 547 556 21428118

[B14] Beccaria M Luisetti M Rodi G Corsico A Zoia MC Colato S Pochetti P Braschi A Pozzi E Cerveri I Long term durable benefit after whole lung lavage in pulmonary alveolar proteinosis Eur Respir J 2004 23 526 531 10.1183/09031936.04.00102704 15083749

[B15] Inoue Y Nakata K Arai T Tazawa R Hamano E Nukiwa T Kudo K Keicho N Hizawa N Yamaguchi E Eda R Oishi K Maeda Y Koreeda Y Kodo N Sakatani M Epidemiological and clinical features of idiopathic pulmonary alveolar proteinosis in Japan Respirology 2006 11 S55 S60 10.1111/j.1440-1843.2006.00810.x 16423273

[B16] Cummings KJ Donat WE Ettensohn DB Roggli VL Ingram P Kreiss K Pulmonary alveolar proteinosis in workers at an indium processing facility Am J Respir Crit Care Med 2010 181 5 458 464 10.1164/rccm.200907-1022CR 20019344 PMC3159086

[B17] Suzuki T Sakagami T Young LR Carey BC Wood RE Luisetti M Wert SE Rubin BK Kevill K Chalk C Whitsett JA Stevens C Nogee LM Campo I Trapnell BC Hereditary pulmonary alveolar proteinosis. Pathogenesis, presentation, diagnosis, and therapy Am J Respir Crit Care Med 2010 182 1292 1304 10.1164/rccm.201002-0271OC 20622029 PMC3001266

[B18] Barilli A Rotoli BM Visigalli R Bussolati O Gazzola GC Kadija Z Rodi G Mariani F Ruzza ML Luisetti M Dall'Asta V In Lysinuric protein Intolerance system y + L activity is defective in monocytes and in GM-CSF-differentiated macrophages Orphanet J Rare Dis 2010 5 32 10.1186/1750-1172-5-32 21110863 PMC2999609

[B19] Ceruti M Rodi G Stella GM Adami A Bolongaro A Baritussio A Pozzi E Luisetti M Successful whole lung lavage in pulmonary alveolar proteinosis secondary to lysinuric protein intolerance: a case report Orphanet J Rare Dis 2007 2 14 10.1186/1750-1172-2-14 17386098 PMC1845139

[B20] Hirakata Y Kobayashi J Sugama Y Kitamura S Elevation of tumour markers in serum and bronchoalveolar lavage fluid in pulmonary alveolar proteinosis Eur Respir J 1995 8 689 696 7656937

[B21] Fujishima T Honda Y Shijubo N Takahashi H Abe S Increased carcinoembryonic antigen concentrations in sera and bronchoalveolar lavage fluids of patients with pulmonary alveolar proteinosis Respiration 1995 626 317 321 8552862 10.1159/000196473

